# Editorial in This Issue

**DOI:** 10.1590/S1677-5538.IBJU.2016.05.01

**Published:** 2016

**Affiliations:** Divisão de Urologia do A.C. Camargo Cancer Center, Fundação A. Prudente, São Paulo, Brasil

The best therapeutic approach of bulbar urethral stenosis is debatable, with several challenging options. In this issue of Int Braz J Urol, we present an editorial (page 868) defending the excision of the stenotic area and the primary anastomotic urethroplasty, authored by Drs. Siegel and Morey, from Dallas, with low complication rates and satisfactory long term results in comparison with the internal urethrotomies or substitutive techniques.

In Difference of Opinion Drs. Da Costa and Guimarães, from AC Camargo Cancer Center, São Paulo Brazil, explain that there are enough scientific evidence favoring the indication of radical prostatectomy in patients with oligometastatic prostate cancer; On the other hand, Drs. Tistau and Smaldone from the Fox Chase Cancer Center, Philadelphia, point that there is no sufficient evidence to metastatic patients be submitted to this surgery, except in clinical trials. To be updated in both these points of view is fundamental to urology community in order to understand the next steps in the therapy of advanced prostatic cancer, which have suffered successive radical transformations in the last years.

A Belgian group reported the results of low dose brachytherapy with I-125, in 274 patients followed by almost 8 years (page 906). The specific survival and biochemical disease control was satisfactory for low and intermediate risk patients, and they found few significant rectal and urinary toxicities. Faimegos et al., from Athens (page 925) reported that high serum levels of free testosterone and bioavailable testosterone were associated in univariate analysis to the diagnostic of prostate cancer in 40 men submitted to a second biopsy, after the first negative biopsy; these data should be tested in large populations in the future.

A binational study from Itajaí, Brazil and Milano, Italy, performed an external validation of the EORTC risk score for recurrence of non-muscle invasive bladder cancer after transurethral resection of the bladder tumor in 205 Brazilian patients (page 932). We must remember that in the Itajaí region (Santa Catarina State), the majority of the population are composed by European descents, with few Afro-Brazilian or Brazilian indigenous; perhaps they present genetics similarities with the European patients. Obesity is known as a risk factor for kidney cancer, but obese patients in some studies do not present unfavorable oncological outcomes after nephrectomy. Focusing possible associations of obesity and renal cell carcinoma, a Turkish group investigate a new concept, the VAI Visceral adipose index, which was associated with higher grades and large size renal tumors (page 955).

Moving to lithiasis, a group from Guangzhou, China, described Its results with minimally invasive percutaneous nephrolithotomy, using ultrasound assisted punctures, in patients with severe spinal cord deformities, without abdominal organs injury (page 960). Researchers from Almeria, Spain proposed the measure in Hounsfield units of the density of the renal papilla in patients with urinary stones, using tomography without contrast. Patients with higher density present high odds to be a stone former patient.

In page 986, we can find quality of life evaluations (performed by phone calls) of women submitted to correction of urinary incontinence surgery (from Austria) and a survey about sexual function of male partners of women with stress urinary incontinence (page 999).

A Canadian group reported the rates of unilateral vas deferens absence in a population of more than 23,000 men submitted to vasectomy and discuss how to conduct these difficult cases (page 1010).

You can also find experimental studies, and surgical videos, case reports, etc.

